# Groundwater Hardness and Alkalinity As Risk Factors for Kidney Stone Disease in Alwar, India: An Ecological Study

**DOI:** 10.7759/cureus.62272

**Published:** 2024-06-12

**Authors:** Souvik Manna, Usharani Rathnam, Arun Udayaraj, Rajesh, Tuhina Shree

**Affiliations:** 1 Community Medicine, All India Institute of Medical Sciences, New Delhi, IND; 2 General Surgery, Employees' State Insurance Corporation (ESIC) Medical College & Hospital, Alwar, IND; 3 Internal Medicine, Employees' State Insurance Corporation (ESIC) Medical College & Hospital, Alwar, IND; 4 Dermatology, Employees' State Insurance Corporation (ESIC) Medical College & Hospital, Alwar, IND; 5 Community Medicine, Employees' State Insurance Corporation (ESIC) Medical College & Hospital, Alwar, IND

**Keywords:** total dissolved solids, kidney stone disease, total alkalinity, hardness, groundwater quality

## Abstract

Introduction: Rajasthan is a semi-arid state in India where people still use groundwater for drinking purposes. However, the quality of groundwater as compared to standards have not been studied in any details. This ecological study was done to study the groundwater quality parameters in the stone-belt states, compare the quality of groundwater in Alwar with the rest of Rajasthan, and study the morbidity profile of surgical in-patients in the same district, with special emphasis on kidney stone disease (KSDs).

Methods: The morbidity profile of patients coming to the surgery department of a tertiary teaching hospital between January 2002 and June 2023 was obtained from the medical records department, and water quality data was obtained from the publicly available Water Resources Information System (WRIS) groundwater dataset for the year 2023. The dataset provided detailed information on the chemical parameters of water samples throughout the country that were evaluated to estimate the quality of groundwater.

Results: It was found that the groundwater in Alwar is non-potable due to the presence of iron, alkalinity, magnesium, and total dissolved solids (TDS). Iron was estimated to be much higher than the acceptable limit of the Bureau of Indian Standards (BIS) drinking-water quality guidelines (0.3 mg/L). Similarly, most of the chemical parameters in the groundwaters of Rajasthan significantly exceeded the national average. The median electrical conductivity, fluoride, magnesium, sodium, hardness, alkalinity, and turbidity were found to be 1680 μS/cm, 1.05 parts per million (PPM), 41 PPM, 233 PPM, 330 PPM, 310 PPM, 988 PPM, respectively, which are above the WHO recommendations for drinking water guidelines.

Conclusions: The levels of iron and total alkalinity were significantly higher in the study district as compared to the rest of the state. Also, magnesium hardness and TDS levels were very high in the groundwater of the entire state of Rajasthan, making the population vulnerable to KSDs in the long run.

## Introduction

Water quality is one of the most important environmental determinants of health and disease. As per the eleventh five-year plan document of India (2007-12), there are about a quarter million quality-affected habitations in the country with more than half affected with excess iron, followed by fluoride, salinity, nitrate, and arsenic in that order [1]. The water quality standards have been prepared by the World Health Organization (WHO), and acceptable limits for contaminants have been determined [2]. It is recommended that the acceptable limit be implemented; however, values in excess of those mentioned under "acceptable" render the water not suitable, but still may be tolerated in the absence of an alternative source but up to the limits indicated under "permissible limit in the absence of alternate source," above which the sources will have to be rejected [1].

The chemical contaminants in water also present well-known health hazards, e.g., skeletal fluorosis, cancers of the gastrointestinal tract, blue baby syndrome, etc. Numerous studies have also highlighted the association of carcinoma gallbladder with high levels of contaminated water by heavy metal exposure like arsenic, especially in the Indo-Gangetic belt [3-5]. However, the evidence linking water quality to health effects, especially kidney stone diseases (KSDs, i.e., nephrolithiasis, ureterolithiasis, and cystolithiasis) is not straightforward [6]. A randomized controlled trial from Europe had shown that hard-water drinking was associated with a higher urinary pH value, being related to the higher bicarbonate water content (HCO_3_^-^ 1,031 mg/l in hard water versus 73 mg/l in soft water [7]. The study concluded that high calcium intake with water between meals increases both the urinary calcium concentration and relative calcium oxalate supersaturation due to unchanged urinary oxalate.

In India, a definitive stone-belt (based on kidney stone burden) has been identified by numerous studies, which extends from the Himalayas to central India, spanning the northern and western states [8]. However, the ecological and environmental determinants which lead to KSD are not well understood. Previous studies from India have shown that drinking less than three liters of water daily can be a risk factor for nephrolithiasis, which has also been substantiated by systematic reviews [6,9]. Other studies have identified some other risk factors, like male gender, increased age, sedentary lifestyles, and diets rich in fats, etc. [10-12]. However, studies on groundwater quality parameters of the stone-belt states are scarce.

The current study was done in one of the semi-arid districts of Rajasthan (Alwar), India where people still use groundwater for drinking purposes. The morbidity profile of patients coming to the surgery department of a tertiary teaching hospital in the region, the Employees' State Insurance Corporation (ESIC) Medical College & Hospital, Alwar, was documented from medical records, and water quality data was obtained from the publicly available Water Resources Information System (WRIS) groundwater dataset. This ecological study was done to study the groundwater quality parameters in the stone-belt states, compare the quality of groundwater in Alwar with the rest of Rajasthan, and study the morbidity profile of surgical in-patients in the same district, with special emphasis on KSDs.

## Materials and methods

Study design

A descriptive cross-sectional study was carried out between January 2022 and June 2023 to describe the morbidity profile of surgical in-patients and to find ecological factors, especially groundwater parameters leading to KSD in the study area. Ethical approval for the study was obtained from the Institute Ethics Committee, Employees' State Insurance Corporation (ESIC) Medical College & Hospital, Alwar (ESIC/MCH/Alwar/2023/IEC/Proj121) and the study followed the tenets of Helsinki Declaration of 1975, as revised in 2000. A total of 310 patients were admitted during the study period, out of which 64 (20.6%) were diagnosed with renal calculi.

Data collection

For the descriptive component, a desk review was done to review the medical records of all patients who were admitted to the general surgery department of the tertiary teaching hospital. The study was conducted in the Alwar district of Rajasthan, which is considered one of the semi-arid districts in the state. The inclusion criteria were in-patient treatment in the surgery ward during the study period irrespective of the diagnosis. The exclusion criteria were out-patient management or day-care treatment, without the need for admission. The hospital medical record department (MRD) used the International Classification of Diseases, Version 10 (ICD-10) codes for reporting the diagnoses. Diagnosis of KSD (ICD10 Code: N20.0) included nephrolithiasis not otherwise specified, renal calculus, renal stone, staghorn calculus, or stone in the kidney. 

The environmental data on quality of groundwater was obtained from publicly available datasets of the Ministry of Jal Shakti, Government of India. The WRIS groundwater dataset for the year 2023 was accessed from the National Data Analytics Platform (NDAP) and visualization was done using the inbuilt tools within the NDAP website [13]. The data was checked for completeness and consistency, and any missing variables or fields were identified. Imputation techniques were used to replace the missing values wherever necessary, and inconsistencies were resolved.

Study tool

A pre-designed questionnaire was used to collect information from the medical records of patients regarding socio-demographic factors, like age, gender, total water consumption, source of water, personal habits, etc. The groundwater data was obtained from the WRIS dataset which was collected by investigators of the Ministry of Jal Shakti, Government of India by taking random samples of groundwater from shallow wells, tube wells, and deep-tube wells located at different places in the country, including the stone-belt area of which Alwar district of Rajasthan is a part.

Variables collected

The groundwater parameters recorded from NDAP WRIS datasets were total carbonate, calcium, chloride, electrical conductivity, fluoride, iron, bicarbonate, potassium, magnesium, nitrate, sodium, sulfate, total hardness, total alkalinity, and total dissolved solids (TDS).

Data analysis

For ecological comparisons, the states were divided into two groups: stone-belt and non-stone-belt states, and the average water quality parameters were compared first between the two groups and then among various stone-belt states themselves. Similarly, water quality indicators of the study area were compared with the remaining districts of the same state. Data were analyzed and compiled using Epi Info statistical software, Version 7.2.1.0 (Centers for Disease Control and Prevention, Atlanta, GA), and IBM SPSS Statistics for Windows, Version 26.0 (IBM Corp., Armonk, NY). Descriptive analysis was performed and tabulated using frequency distribution tables and proportions. The non-parametric Mann-Whitney U test was used to compare the mean water quality parameters, and the level of significance was set at <0.05.

## Results

A total of 310 patients were admitted to the surgery ward from January 2022 to June 2023. The mean age of the admitted patients was 41.3±10.1, with a range of 21 to 59 years. There was no significant difference in the ages of male and female participants (41.3±10.5 versus 42.5±9.3 respectively). Most of the study participants belonged to the 31 to 50 years age range, with males constituting more than half of the total participants, i.e., 145 (57.3%).

The morbidity profile of the study participants revealed that major gastrointestinal (GI) problems leading to hospitalization were gastritis (57, 18.39%), hernia (56, 18.06%), gall bladder stones (52, 16.8%), and appendicitis (26, 8.36%) (Table 1). Similarly, KSD was the major renal disorder leading to hospitalizations (64, 20.65%), liver abscess (3, 0.97%) was the major hepato-biliary cause, lipoma (9, 2.90%) was the main neoplastic condition, and epididymo-orchitis (16, 5.16%) was the primary genito-urinary disorder leading to hospitalization (Table 1).

**Table 1:** Morbidity profile of the in-patients admitted to the surgery ward (N=310). AGE: Acute gastroenteritis, PID: Pelvic inflammatory disease, UTI: Urinary tract infection, BPH: Benign prostatic hypertrophy. * Others include gastric outlet obstruction (2, 0.65%), dysphagia (2, 0.65%), cholecystitis (2, 0.65%), constipation (2, 0.65%), diverticulitis (1, 0.32%), rectal polyp (1, 0.32%), peptic ulcer disease (1, 0.32%), and gastroesophageal reflux disease (GERD) (1, 0.32%). † Others include hydronephrosis (2, 0.65%), renal colic (1, 0.32%), neurogenic bladder (1, 0.32%), cystitis (1, 0.32%), vesical calculus (2, 0.65%), horseshoe kidney (1, 0.32%), urinary retention (1, 0.32%), priapism (1, 0.32%), ureteric calculi (1, 0.32%), renal agenesis (1, 0.32%), ectopic kidney (3, 0.97%), pyelonephritis (1, 0.32%), cortical renal cyst (2, 0.65%), and pelvi-ureteric junction (PUJ) stent (1, 0.32%). ‡ Others include cholangitis (1, 0.32%), liver cirrhosis (1, 0.32%), obstructive jaundice (1, 0.32%), alcoholic hepatitis (1, 0.32%), and hepatomegaly (2, 0.65%). § Others include pleomorphic adenoma (1, 0.32%), thyroid nodule (1, 0.32%), secondaries in the liver (1, 0.32%), and mucinous retention cyst (1, 0.32%). || Others include acute cystitis (1, 0.32%), funiculitis (1, 0.32%), orchitis (2, 0.65%), and pyocele (1, 0.32%).

Categories	Diagnosis	N (%)
Gastrointestinal tract	Gastritis	57 (18.39)
	Hernia	56 (18.06)
	Cholelithiasis	30 (9.68)
	Appendicitis	26 (8.36)
	Choledocholithiasis	22 (7.10)
	Hemorrhoids	18 (5.81)
	Colitis	17 (5.48)
	Fissure in ano	13 (4.19)
	Pancreatitis	11 (3.55)
	Appendicular lump	10 (3.23)
	Pain abdomen	9 (2.90)
	Perianal abscess	6 (1.94)
	Intestinal obstruction	4 (1.29)
	Umbilical hernia	3 (0.97)
	AGE/diarrhoea	3 (0.97)
	Others*	12 (3.98)
Kidney	Renal calculi	64 (20.65)
	PID	15 (4.84)
	UTI	7 (2.26)
	Others †	19 (6.13)
Hepatobiliary	Liver abscess	3 (0.97)
	Others ‡	6 (1.94)
Breast	Fibroadenoma	3 (0.97)
	Mastitis	2 (0.65)
Ulcers	Non-healing ulcer	3 (0.97)
Tumors	Lipoma	9 (2.90)
	Ovarian hemorrhagic cyst	2 (0.65)
	Pseudocyst pancreas	2 (0.65)
	Others §	4 (1.29)
Metabolic	Diabetes	4 (1.29)
Genito-urinary	Epididymo-orchitis	16 (5.16)
	Hydrocele	11 (3.55)
	BPH	9 (2.90)
	Others ||	5 (1.61)
Skin	Sebaceous cyst	4 (1.29)
	Dermoid cyst	2 (0.65)
	Cellulitis	2 (0.65)
	Pilonidal sinus	2 (0.65)
	Epidermoid cyst	1 (0.32)
	Finger abscess	1 (0.32)

**Figure 1:**
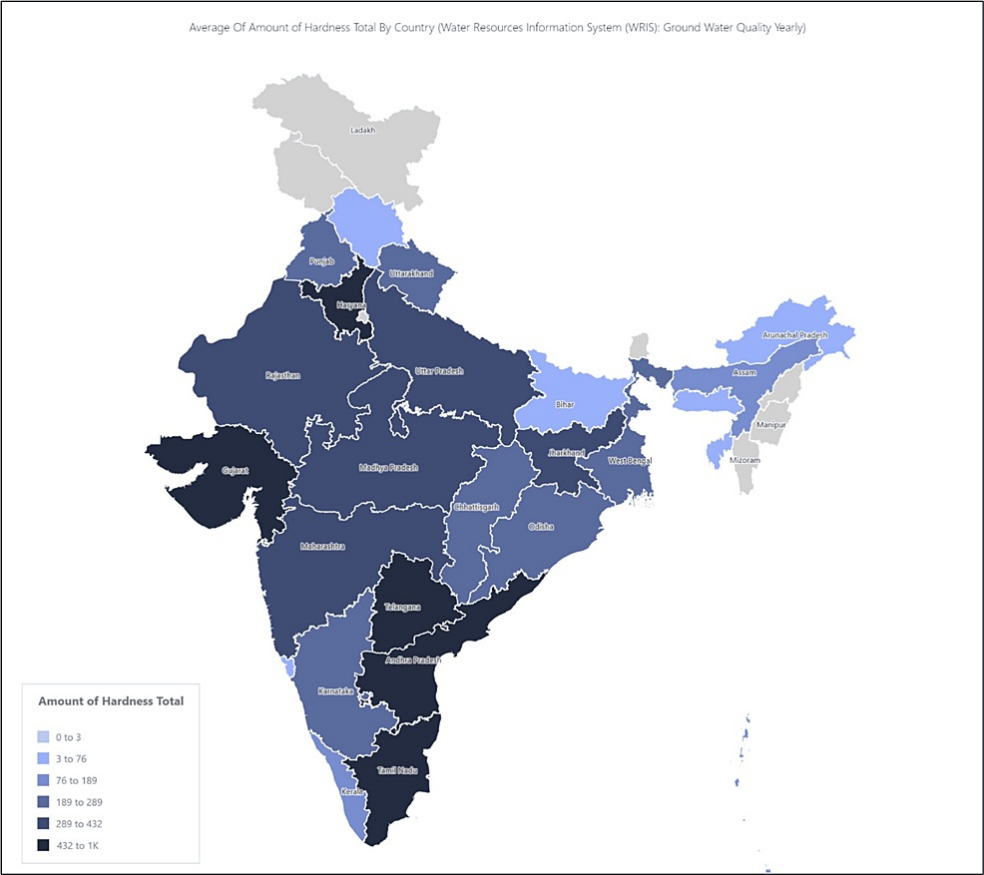
Map of India showing the stone-belt states. WRIS: Water Resources Information System. The amount of total hardness is the sum of calcium and magnesium hardness, expressed as carbonates in parts per million (PPM). The figure was originally created by the authors from data uploaded by the Ministry of Jal Shakti, Government of India on the National Data Analytics Portal.

**Figure 2:**
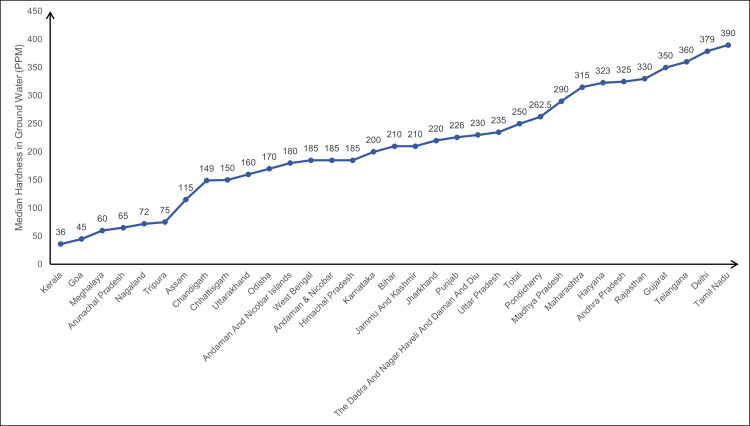
Median hardness in groundwater in various states of India. PPM: Parts per million. The figure was originally created by the authors from data uploaded by the Ministry of Jal Shakti, Government of India on the National Data Analytics Portal.

The environmental determinants were mainly groundwater quality parameters and have been recorded for stone-belt states. The stone-belt states are those that have higher levels of hardness and TDS in the groundwater (Figures 1, 2).

These states are Tamil Nadu, Telangana, and Andhra Pradesh in the south; Gujarat, Maharashtra, and Rajasthan in the west; Madhya Pradesh in the center; Punjab, Delhi, and Haryana in the north; and Bihar and West Bengal in the east. The median values of the groundwater parameters in these states were compared with the WHO standards for drinking water quality [2,14]. In case, the units were not comparable, mEq/L were converted to parts per million (PPM) using the formula PPM=mEq/L x (molecular weight/valency) [15]. The groundwater quality was assessed with respect to various parameters, like calcium, magnesium, chloride, fluoride, iron, electrical conductivity, TDS, etc., as mentioned in the Methods section.

Calcium

The acceptable limit of calcium is 75 PPM, and the permissible limit is 200 PPM. None of the stone-belt states had median calcium levels exceeding the acceptable limits, with wide variation in ranges (Table 2). As far as Rajasthan is concerned, the median calcium level in groundwater was 60 PPM (Range: 2-2800), and the mean calcium was 80.1 (±90.4) PPM. Nearly, 6624 (63.5%) of the habitations had normal calcium levels (≤75 PPM), 3161 (30.3%) had calcium levels exceeding acceptable levels but within permissible limits (76-200 PPM), while 644 (6.2%) habitations had groundwater calcium above the permissible limit of 200 PPM. In the Alwar district from where the renal calculi cases were studied, the median calcium level was 44 (Range: 4-580), and the mean was 62.8 (±68.9) PPM. A total of 333 (77.6%) habitations had normal, 81 (18.9%) had more than acceptable levels, and 15 (3.5%) had more than permissible levels of calcium in the groundwater.

**Table 2:** Comparison of median (with range) groundwater quality parameters among stone-belt states (Ministry of Jal Shakti). * As per IS 10500-2012, by the Bureau of Indian Standards. WHO GV: World Health Organization Guideline Value, NA: No data available, PPM: Parts per million, EC: Electrical conductivity, μS/cm: microsiemens per centimeter, HCO_3_^-^: Bicarbonate anion, K^+^: Potassium ion,  Mg_2_^+^: Magnesium ion, SO_4_^2-^: Sulfate anion, TDS: Total dissolved solids. Source of data: Water Resources Information System, Ministry of Jal Shakti, Government of India through the National Data Analytics Portal.

Indicator	Rajasthan	Maharashtra	Gujarat	Punjab	Haryana	Delhi	Madhya Pradesh	Acceptable*	WHO GV
Calcium (PPM)	60 (0-2800)	52 (2-817.63)	68 (2-1980)	35 (2-650)	41 (0-886)	71 (6.4-1584)	64 (0-856)	75 PPM	250 PPM
Chloride (PPM)	220 (7-20,000)	73.20 (1.24-7461)	184 (7-16330)	50 (2.8-4417)	159.5 (1.3-18100)	203 (6.7-8710)	60 (2-3716)	250 PPM	NA
EC (μS/cm)	1680 (120-75000)	868 (27-29370)	1350 (71-48200)	896 (129-41410)	1520 (62-42700)	1610 (197-25300)	845 (10-33800)	1000	1000 μS/cm
Fluoride (PPM)	1.05 (0-65.51)	0.36 (0-57)	0.6 (0-12)	0.47 (0-22.6)	0.8 (0-32)	0.75 (0.09-15.9)	0.43 (0-12.6)	1.0 PPM	1.5 PPM
Iron (PPM)	0.25 (0-33)	0.09 (±0.38)*	0.15 (0-26)	0.07 (0-16.85)	0.03 (0-12.95)	NA	0.23 (±0.78)	0.3 PPM	NA
HCO_3_^- ^(PPM)	366 (2.32-9650)	268 (0-2313.58)	317 (1.9-3050)	317 (0-2489)	309 (13-1757)	323 (38-1291)	287 (0-2658)	NA	NA
K^+ ^(PPM)	5 (0-944)	2 (0.01-450)	2.9 (0-1110)	7.5 (0-1000)	7.6 (0-1100)	5.8 (0.2-395)	1.5 (0-430)	12 PPM	NA
Mg^2+ ^(PPM)	41 (0.07-1209)	41 (0.03-1051.57)	41.37 (0-1592)	32.65 (0-703)	52 (0-1203.31)	46 (1.02-979)	27 (67-593.24)	30 PPM	150 PPM
Nitrate (PPM)	34 (0-4405)	27 (0-2730)	26 (0-1600)	22 (0-2223)	19 (0-3500)	25 (0.03-1500)	25 (0-1000)	45 PPM	50 PPM
Sodium (PPM)	233 (2.35-9750)	38 (0-8087)	140 (2-8050)	95 (1.4-3118)	175 (1-5800)	192.5 (1.8-3650)	49 (0-1890)	20 PPM: Na_2_CO_3_, 150 PPM: NaCl	
SO_4_^2- ^(PPM)	118 (0-8862)	36 (0-3900)	52 (0-9332)	67 (0-5000)	146 (0-6560)	134.5 (2-5000)	30 (0-3200)	200 PPM	NA
Hardness (PPM)	330 (10-9445)	315 (10-4625)	350 (20-8700)	226 (0-3878)	323 (0-6546)	379 (60-6775)	290 (6-3100)	200 PPM	500 PPM
Alkalinity (PPM)	309.8 (1.9-7909.8)	185.3 (0-1345.1)	259.84 (1.6-2500)	254.9 (0-1622)	240.4 (0-1316)	269.7 (31.2-1238.2)	209.84 (0-2178.7)	200 PPM	NA
TDS (PPM)	988 (0-31525)	314.32 (±517.68)	901.15 (0-17809)	NA	NA	NA	259 (±395.19)	500 PPM	1000 PPM

Chloride

A total of 10425 habitations were selected in Rajasthan for the collection of groundwater samples for chloride level estimation, as per the WRIS database. The median chloride level in groundwater samples was 220 (Range: 7-20,000) and the mean was 487.8 (±785.7). Nearly half of the habitations had normal chloride levels (5605, 53.8%), i.e., less than 250 PPM. Around one-third (3464, 33.2%) of households had levels exceeding the acceptable level of 250 PPM but within the permissible limit of 1000 PPM, whereas 1356 (13.0%) habitations had non-permissible levels of chloride in drinking water. In Alwar district, a total of 429 (4.1%) habitations were situated. The median and mean chloride levels were lesser compared to the state averages, with 284 habitations showing normal levels (66.2%), 112 (26.1%) exceeding acceptable limits, and 33 (7.7%) habitations with non-permissible levels.

Fluoride

All other stone-belt states had median fluoride levels within the acceptable range of 1-1.5 PPM, except Rajasthan. Out of the 10419 habitations tested for groundwater fluoride levels in Rajasthan, more than half had unacceptable levels, 1957 (18.8%) crossing the 1 PPM acceptable limit and 3399 (32.6%) crossing the 1.5 PPM permissible limit. In Alwar, the mean and median fluoride levels were within normal limits, however, 81 (18.9%) and 109 (25.4%) habitations out of a total of 429 had fluoride levels above acceptable and permissible limits respectively.

Iron

The level of iron in drinking water should not exceed 0.3 PPM, and the acceptable and permissible limits are the same. Excess iron is generally not a cause of health concern but may cause acceptability issues. The number of habitations checked for iron in Rajasthan was 9670, out of which 4326 (44.7%) had levels exceeding the 0.3 PPM cut-off. The median and mean were 0.25 (Range: 0-33) and 0.8 (±1.6) respectively. In Alwar, the median was 0.3 (Range: 0.004-18.6), and nearly half of the habitations had unacceptable levels (202, 49.4%).

Magnesium

Six of the nine stone-belt states studied had levels of magnesium more than the acceptable level of 30 PPM but within the permissible limit of 100 PPM. In Rajasthan, the median magnesium level was 41 PPM (Range: 0.07-1209), and the mean was 64.5 PPM (±74.8), however, nearly half of the habitations (5185, 49.7%) had levels above acceptable limits, and 1696 (16.3%) of the total 10430 habitations were having non-permissible levels. In Alwar, nearly three-fifths of the habitations had more than acceptable levels of magnesium in groundwater samples.

Nitrates

The cut-off level of nitrates is 45 PPM, with no relaxation for permissibility. In Rajasthan and Alwar, nearly 40% and 30% of habitations respectively exceeded the cut-off level of 45 PPM.

Sulfates

The drinking standards laid down by the WHO are 250 mg/L of sulfates and the maximum admissible concentration can reach 400 mg/L if magnesium does not exceed 30 PPM [16].

The Indian standards stipulate 200 PPM as the acceptable limit, and none of the stone-forming states surpassed that limit, with wide variation in different geographical areas. In Rajasthan and Alwar, nearly 35% and 20% of habitations respectively exceeded the acceptable level of sulfates.

Alkalinity: carbonate and bicarbonates

As for bicarbonates, the presence of HCO_3_^−^ ions in water gives it a pleasant odor and does not present any risk to human health. According to the Indian standard code for drinking water, IS 10500-2012, the acceptable limit of total alkalinity is 200 mg/L and the permissible limit is 600 PPM [1]. In Rajasthan, including Alwar, the mean alkalinity was 309.8 (Range: 1.9-7909.8) and nearly four-fifths of the habitations (7895, 80.7%) exceeded the acceptable limits.

Sodium and potassium

The guideline value for sodium chloride is 150 PPM, and 250 PPM is the upper limit for taste threshold, as per WHO. Out of 10430 groundwater samples from Rajasthan, 1603 (15.4%) had levels exceeding 150 PPM, and 4940 (47.4%) exceeded 250 PPM. The median and mean were 233 (Range: 2.35-9750) and 400.3 (±511.2) respectively. The water quality of Alwar was slightly better than the rest of Rajasthan, but nearly 50% of the habitations had brackish groundwater. 

As far as potassium is concerned, the levels were within permissible limits of 12 PPM in all the states.

Electrical conductivity (EC)

According to WHO standards, EC values above 1000 μS/cm are not acceptable, but values up to 2500 μS/cm are reported in some guidelines as permissible. In Rajasthan, the median EC was 1680 μS/cm (Range: 120-75000) and the mean was 2524.5 μS/cm (±2674.8). Nearly two-fifths of the habitations (4117, 39.5%) exceeded acceptable limits, and 3548 (34.0%) exceeded permissible limits.

Total dissolved solids

As per BIS, the acceptable and permissible limits of TDS are 500 PPM and 2000 PPM respectively. The median TDS level of ground waters in Rajasthan was 988 (Range: 0-31525) and the mean was 1517.2 (±1726.5), clearly exceeding the acceptable limits. Only 376 (19.1%) of the habitations had normal TDS levels, while the remaining 80% of the total 1965 habitations tested for TDS in Rajasthan were abnormal.

The mean water quality parameters of Rajasthan were compared with the rest of the country using the Mann-Whitney U test and were found to be significantly higher in all the parameters. The statistical significance is due to the very large sample size in both groups, about 10430 habitations in Rajasthan and 0.1 million in the rest of the country (Table 3). However, what is more remarkable than the difference, is the proportion of habitations in Rajasthan which surpass the acceptable and permissible limits of drinking water quality parameters, and fall in the risky level for health issues. Of course, not all households consume groundwater directly, but those that do will be highly at risk of adverse health outcomes.

**Table 3:** Comparison of mean groundwater quality parameters between Rajasthan and all-India average (Ministry of Jal Shakti). *Statistically significant difference in Mann-Whitney U test for non-parametric data (p value <0.05). EC: Electrical conductivity, CO_3_^2-^: Carbonate, Z: Z statistic, SD: Standard deviation, PPM: Parts per million, μS/cm: microsiemens per centimeter, HCO_3_^-^: Bicarbonate anion, K^+^: Potassium ion,  Mg_2_^+^: Magnesium ion, SO_4_^2-^: Sulfate anion, TDS: Total dissolved solids.

Parameter	Rajasthan		Number of habitations, N=10430			All India (except Rajasthan)	N	Z	p value
	Median (Range)	Mean (± SD)	Normal	>Acceptable	>Permissible				
Calcium (PPM)	60 (2-2800)	80.1 (±90.4)	6624 (63.5)	3161 (30.3)	644 (6.2)	61.3 (±67.6)	107131	-32.7	0.000*
Chloride (PPM)	220 (7-20,000)	487.8 (±785.7)	5605 (53.8)	3464 (33.2)	1356 (13.0)	171.5 (±385.8)	119080	-79.1	0.000*
EC (μS/cm)	1680 (120-75000)	2524.5 (±2674.8)	2767 (26.5)	4117 (39.5)	3548 (34.0)	1131.3 (±1413.8)	129750	-88.4	0.000*
Fluoride (PPM)	1.05 (0-65.51)	1.5 (±2.2)	5063 (48.6)	1957 (18.8)	3399 (32.6)	0.6 (± 0.9)	118284	-88.2	0.000*
Iron (PPM)	0.25 (0-33)	0.8 (±1.6)	5344 (55.3)	4326 (44.7)	4326 (44.7)	0.7 (± 0.9)	28896	-39.0	0.000*
HCO_3_^- ^(PPM)	366 (2.32-9650)	416.8 (±257.7)	1371 (13.1)	7381 (70.8)	1676 (16.1)	273.9 (±183.2)	104538	-67.4	0.000*
K^+^ (PPM)	5 (0-944)	20.1 (±56.8)	7674 (74.5)	2622 (25.5)	2622 (25.5)	15.2 (±47.6)	99357	-30.9	0.000*
Mg^2+ ^(PPM)	41 (0.07-1209)	64.5 (±74.8)	3549 (34.0)	5185 (49.7)	1696 (16.3)	40.8 (±60.9)	107751	-52.8	0.000*
Nitrate (PPM)	34 (0-4405)	81.8 (±144.9)	6134 (58.8)	4291 (41.2)	4291 (41.2)	45.7 (±89.0)	103764	-42.0	0.000*
Sodium (PPM)	233 (2.35-9750)	400.3 (±511.2)	3887 (37.3)	1603 (15.4)	4940 (47.4)	118.1 (±224.9)	100801	-97.3	0.000*
SO_4_^2- ^(PPM)	118 (0-8862)	232.9 (±376.6)	6941 (66.6)	1872 (17.9)	1613 (15.5)	83.8 (±191.3)	97739	-82.8	0.000*
Hardness (PPM)	330 (10-9445)	462.9 (±467.3)	2257 (21.6)	6094 (58.4)	2079 (19.9)	315.2 (±335.9)	109220	-48.7	0.000*
Alkalinity (PPM)	309.8 (1.9-7909.8)	355.9 (±215.9)	1884 (19.3)	6929 (70.9)	966 (9.9)	214.1 (±162.7)	66442	-74.9	0.000*
TDS (PPM)	988 (0-31525)	1517.2 (±1726.5)	376 (19.1)	1144 (58.2)	445 (22.6)	442.5 (±802.6)	17692	-50.4	0.000*

Similarly, the mean parameters of Alwar were compared with the rest of Rajasthan using non-parametric tests, and a statistically significant increase was found in groundwater iron and total alkalinity, with no difference in magnesium and TDS levels (Table 4).

**Table 4:** Comparison of water quality parameters of Alwar district with the rest of Rajasthan. *Statistically significant difference found in Mann-Whitney U test for parametric data (p value <0.05). NA: No data available, EC: Electrical conductivity, CO_3_^2-^: Carbonate, Z: Z statistic, SD: Standard deviation, PPM: Parts per million, μS/cm: microsiemens per centimeter, HCO_3_^-^: Bicarbonate anion, K^+^: Potassium ion, Mg_2_^+^: Magnesium ion, SO_4_^2-^: Sulfate anion, TDS: Total dissolved solids.

Parameter	Locality	Median (Range)	Mean (± SD)	Normal	>Acceptable	>Permissible	Total	Z value	p value
Calcium (PPM)	Rajasthan	60 (2-2800)	80.1 (±90.4)	6291 (62.9)	3080 (30.8)	629 (6.3)	10000	-6.7	0.000*
	Alwar	44 (4-580)	62.8 (±68.9)	333 (77.6)	81 (18.9)	15 (3.5)	429		
Chloride (PPM)	Rajasthan	220 (7-20,000)	487.8 (±785.7)	5321 (53.2)	3352 (33.5)	1323 (13.2)	9996	-7.6	0.000*
	Alwar	121 (14-3550_	325.5 (±536.3)	284 (66.2)	112 (26.1)	33 (7.7)	429		
EC (μS/cm)	Rajasthan	1680 (120-75000)	2524.5 (±2674.8)	2605 (26.0)	3948 (39.5)	3450 (34.5)	10003	-5.9	0.000*
	Alwar	1290 (272-23140)	2057.2 (±2374.3)	162 (37.8)	169 (39.4)	98 (22.8)	429		
Fluoride (PPM)	Rajasthan	1.1 (0-65.51)	1.5 (±2.2)	4824 (48.3)	1876 (18.8)	3290 (32.6)	9990	-4.2	0.000*
	Alwar	0.9 (0.01-25)	1.4 (±1.9)	239 (55.7)	81 (18.9)	109 (25.4)	429		
Iron (PPM)	Rajasthan	0.25 (0-33)	0.8 (±1.6)	5137 (55.5)	4124 (44.5)	NA	9261	-2.8	0.006*
	Alwar	0.3 (0.004-18.6)	1.01 (±1.9)	207 (50.6)	202 (49.4)	NA	409		
K^+ ^(PPM)	Rajasthan	5 (0-944)	20.1 (±56.8)	7280 (73.7)	2596 (26.3)	NA	9876	-12.7	0.000*
	Alwar	2.6 (0.1-920)	9.5 (±64.8)	394 (93.8)	26 (6.2)	NA	420		
Mg^2+^(PPM)	Rajasthan	41 (0.07-1209)	64.5 (±74.8)	3418 (34.2)	4939 (49.4)	1644 (16.4)	10001	-0.77	0.939
	Alwar	39 (4.9-771)	65.4 (±90.5)	131 (30.5)	246 (57.3)	52 (12.1)	429		
Nitrate (PPM)	Rajasthan	34 (0-4405)	81.8 (±144.9)	5834 (58.4)	4162 (41.6)	NA	9996	-4.6	0.000*
	Alwar	25 (0.3-884.2)	55.1 (±84.9)	300 (69.9)	129 (30.1)	NA	429		
Sodium (PPM)	Rajasthan	233 (2.35-9750)	400.3 (±511.2)	3689 (36.9)	1535 (15.4)	4777 (47.8)	10001	-4.9	0.000*
	Alwar	175 (16-3404)	298.2 (±392.7)	198 (46.2)	68 (15.9)	163 (37.9)	429		
SO_4_^2- ^(PPM)	Rajasthan	118 (0-8862)	232.9 (±376.6)	6597 (65.9)	1829 (18.3)	1571 (15.7)	9997	-7.8	0.000*
	Alwar	64 (2-6225)	187.7 (±554.2)	344 (80.2)	43 (10.0)	42 (9.8)	429		
Hardness (PPM)	Rajasthan	330 (10-9445)	462.9 (±467.3)	2170 (21.7)	5805 (58.0)	2026 (20.3)	10001	-3.4	0.001*
	Alwar	290 (50-4500)	425.1 (±516.6)	87 (20.3)	289 (67.4)	53 (12.4)	429		
Alkalinity (PPM)	Rajasthan	309.8 (1.9-7909.8)	355.9 (±215.9)	1829 (19.5)	6643 (70.7)	922 (9.8)	9394	-3.4	0.001*
	Alwar	330.3 (6.1-1140.2)	376.6 (±179.0)	55 (14.3)	286 (74.3)	44 (11.4)	385		
TDS (PPM)	Rajasthan	988 (0-31525)	1517.2 (±1726.5)	356 (19.1)	1077 (57.8)	431 (23.1)	1864	-1.8	0.077
	Alwar	799.5 (191.8-15041)	1333.8 (±1730.6)	20 (19.8)	67 (66.3)	14 (13.9)	101		

## Discussion

The biological hazards of drinking water contaminated with micro-organisms are well documented. However, chemicals and metals present in drinking water constitute an important chemical hazard over chronic or lifetime exposure to polluted waters. The current study was done to compare the groundwater parameters of stone-belt states, including Rajasthan, with national and international standards, and comment on the probable association with KSD in one high-risk district of Rajasthan.

The current study found that the level of nitrates was above the permissible limits in one-third of sites in Rajasthan. A study from adjoining Dausa district of Rajasthan reported that 28% of the groundwater samples tested for nitrate were beyond the permissible limit of 45 mg/L as per the BIS limits [17]. The reasons for this increase are numerous, including the use of nitrate fertilizers, emphasis on nitrate-dense crops, and seepage of domestic and industrial sewage into groundwater. This is also alarming given the proven association of nitrates with adverse health effects like methemoglobinemia in bottle-fed infants (blue-baby syndrome), colorectal cancer, thyroid disease, and neural tube defects [18]. 

Endemic fluorosis leading to skeletal changes in adults after chronic exposure to water high in fluorides is well documented in Rajasthan [19-21]. The WHO guideline value of 1.5 PPM fluoride in drinking water has been made more stringent by the Bureau of Indian Standards (BIS) to 1 PPM. This is due to the increased volumes of water consumed by Indians during summer and intake from other sources, like food. Nearly half of the sites in Rajasthan had groundwater levels above the 1 PPM limit. In Rajasthan, brackish water (>150 PPM NaCl) is a common problem due to the dissolution of salts from the surrounding rocks and soil, where adequate sources of surface water are not available. In the current study, brackish groundwater reserves were found in nearly 60% of the habitations. Although several studies suggest that high levels of sodium in drinking water are associated with increased blood pressure in children, in other studies no such association has been found [22,23]. On the other hand, potassium deficiency is rarely found anywhere but may lead to depression, muscle weakness, heart rhythm disorder, etc. [24].

Groundwater chloride was found to be satisfactory in most of the habitations. However, this should not be confused with free residual chlorine, which has an acceptable limit of only 0.2 mg/L (PPM) and is applicable only when water is chlorinated and tested at the consumer end. When protection against viral infection is required, free chlorine should be a minimum of 0.5 mg/L [25].

The calcium concentration of water has been documented to range from 1 to 135 mg/L across North America [26]. However, the WHO standard for drinking water has set a maximum permissible limit of 250 PPM for calcium hardness, 150 PPM, for magnesium hardness, 500 PPM for total harness, 1000 PPM for TDS, and 1200 PPM for electrical conductance [27]. Generally speaking, the upper limit of chloride (as Cl−) concentrations in freshwater is considered to be 200 mg/L (0.2%) [28].

The current study found that Rajasthan has much higher levels of groundwater cations and anions as compared to the national average. However, the Alwar district has much higher levels of iron and total alkalinity in groundwater. Iron is not hazardous to health, but it is considered a secondary or aesthetic contaminant, leaving reddish brown stains on fixtures, tableware, and laundry that are very hard to remove.

The combination of carbonate, bicarbonate, and hydroxide anions gives the alkalinity of water. According to the Indian standard code for drinking water, IS 10500-2012, the acceptable limit of total alkalinity is 200 mg/L and the permissible limit is 600 PPM [1]. However, most of the stone-forming states in western, northern, and central India, except Maharashtra had total alkalinity levels more than the permissible limits. This is a serious cause for concern for patients who are prone to KSD. Previous studies have shown that drinking bicarbonate alkaline water with a high calcium content is significantly lithogenic [29].

The TDS level in water represents the total amount of inorganic (e.g., potassium, calcium, sodium, bicarbonates, chlorides, magnesium, sulfates) and organic minerals present in water. TDS testing is based on conductivity and is expressed in parts per million (PPM) or milligrams per liter (mg/L). According to the Environmental Protection Agency (EPA), the maximum contaminant level (MCL) of TDS for human consumption is 500 PPM [30]. A very alarming finding in the current study was that nearly 80% of the habitations exceeded the 500 PPM limit for TDS. Although the relationship between TDS in drinking water and KSD is not straightforward, the current study clearly provides ecological evidence of an association between the two.

The strengths of the current study are a large sample size and a comprehensive analysis of multiple groundwater quality parameters across different regions. The publicly available data provides a rich mine of information from which patterns and trends of disease can be identified, especially those related to groundwater. The only bottleneck is the lack of comprehensive reporting of diseases by the hospitals. The current study also had some limitations. Firstly, a cross-sectional ecological study can not provide causal associations between exposure and disease, because individual-level exposures are not measured. Second, secondary data collected from publicly available resources do not specify the objectives and methodology used to collect the environmental samples. A focused research study to identify the environmental determinants of KSD can provide a much higher level of evidence, as compared to the present study. The important bias in any ecological study is an ecological fallacy, in which group differences are attributed to individual-level exposures, without taking into account the intrinsic variability among human communities.

## Conclusions

The study concludes that an ecological link exists between the presence of kidney stone disease and the amount of hardness, alkalinity, and total dissolved solids in drinking water. Also, the groundwater in most of the stone-belt states is lithogenic and hence is unsuitable for consumption. The study cannot ascertain causal associations because it is ecological in nature. However, it has implications for policy, because it identifies an environmental risk factor to which millions of people are exposed daily. The quality of groundwater in stone-belt states has not been studied in any detail, and the current study opens an avenue for further research exploring the chronic health effects of drinking groundwater contaminated with chemicals and heavy metals.
